# Trends and Characteristics of Manufactured Cannabis Product and Cannabis Plant Product Exposures Reported to US Poison Control Centers, 2017-2019

**DOI:** 10.1001/jamanetworkopen.2021.10925

**Published:** 2021-05-24

**Authors:** Julia A. Dilley, Janessa M. Graves, Ashley Brooks-Russell, Jennifer M. Whitehill, Erica L. Liebelt

**Affiliations:** 1 Oregon Public Health Division, Portland, Oregon; 2 Washington State University, Spokane; 3 University of Colorado Anschutz Medical Campus, Aurora; 4 University of Massachusetts Amherst, Amherst; 5 Washington Poison Center, Seattle, Washington; 6 Now at Department of Pediatrics, Section of Pediatric Emergency Medicine, Pharmacology & Toxicology, College of Medicine, University of Arkansas for Medical Sciences, Little Rock

## Abstract

This cross-sectional study examines reports of cannabis exposure at US poison control centers for trends in patient and product characteristics, stratified by manufactured cannabis products and plant materials.

## Introduction

Legalization of adult use cannabis products has led to a consumer-driven marketplace. A growing share of retail cannabis sales are manufactured cannabis products,^1^ which may contain higher levels of tetrahydrocannabinol (THC) than unprocessed cannabis plant materials,^2^ resulting in greater short-term effects (eg, cognitive and psychomotor impairment).^3^ Public health concerns are emerging about these risks.^4^ Our study objective was to assess recent patterns in reports of cannabis-related exposures by product type.

## Methods

For this cross-sectional study, we accessed National Poison Data System data on cannabis exposures reported to US poison centers for January 2017 through December 2019. Cannabis product type codes were added to the system in 2016. We compared trends and characteristics of exposures for manufactured products that require processing of plant materials (eg, concentrates, edibles, vaporized liquids) and unprocessed plant materials (eg, flower) (eMethods in the Supplement). Two-sided *P* values <.05 were considered statistically significant. This study followed the Strengthening the Reporting of Observational Studies in Epidemiology (STROBE) reporting guideline for cross-sectional studies. The institutional review board at Washington State University determined this study was exempt from review because it used deidentified data.

## Results

Among the total 28 630 exposures, plant materials were the most commonly involved (18 763 exposures [65.5%]), followed by edibles (5537 [19.3%]), concentrates (2734 [9.6%]), vaporized liquids (1075 [3.8%]), and other manufactured products (521 [1.8%]) (Table).

**Table.** Characteristics of Cannabis-Related Exposures Reported to US Poison Centers by Cannabis Product Type, January 2017 to December 2019

Characteristic	Exposures, No. (%)						*P* value
	Specific manufactured products				General product categories		
	Concentrates (n = 2734)	Vaporized liquids (n = 1075)	Edibles (n = 5537)	Other (n = 521)	Total manufactured (n = 9867)	Plant materials (n = 18 763)	
Total							<.001
2017	275 (10.1)	33 (3.1)	697 (12.6)	89 (17.1)	1094 (11.1)	7146 (38.1)	
2018	1002 (36.7)	151 (14.1)	1943 (35.1)	174 (33.4)	3270 (33.1)	6011 (32.0)	
2019	1457 (53.3)	891 (82.9)	2897 (52.3)	258 (49.5)	5503 (55.8)	5606 (29.9)	
Age group							<.001
Children (<10 y)	423 (16.6)	77 (7.5)	1905 (36.6)	100 (20.3)	2505 (27.0)	1490 (8.4)	
Youth (10-20 y)	999 (39.1)	551 (53.6)	1561 (30.0)	95 (19.3)	3206 (34.5)	7.369 (41.3)	
Adults (≥21 y)	1134 (44.4)	400 (38.9)	1746 (33.5)	297 (60.4)	3577 (38.5)	8.984 (50.4)	
Caller site							<.001
Health care facility	1375 (50.3)	644 (59.9)	2676 (48.3)	248 (47.6)	4943 (50.1)	13 624 (72.6)	
Residence	1100 (40.2)	373 (34.7)	2230 (40.3)	224 (43.0)	3927 (39.8)	3817 (20.3)	
Other	243 (8.9)	52 (4.8)	570 (10.3)	47 (9.0)	912 (9.2)	1206 (6.4)	
Unknown	16 (0.6)	6 (0.6)	61 (1.1)	2 (0.4)	85 (0.9)	115 (0.6)	
Coingestants							<.001
Cannabis only	2138 (78.2)	633 (58.9)	4898 (88.5)	371 (71.2)	8040 (81.5)	7207 (38.4)	
Polysubstances	596 (21.8)	442 (41.1)	639 (11.5)	150 (28.8)	1827 (18.5)	11 556 (61.6)	
Medical outcome^a^							.001
Minor or less	1369 (64.0)	365 (57.7)	3114 (63.6)	274 (73.9)	5122 (63.7)	4404 (61.1)	
Moderate or greater	769 (36.0)	268 (42.3)	1784 (36.4)	97 (26.2)	2918 (36.3)	2803 (38.9)	
Legal status of state (2019 exposures only)^b^							<.001
Not legal	946 (65.4)	692 (78.0)	1328 (46.5)	153 (59.3)	3119 (57.3)	3806 (68.7)	
Rate per 100 000	0.40	0.29	0.56	0.07	1.33	1.62	
Adult use legal	501 (34.6)	195 (22.0)	1528 (53.5)	105 (40.7)	2329 (42.8)	1735 (31.3)	
Rate per 100 000	0.54	0.21	1.64	0.11	2.50	1.86	
Quarterly slope coefficients (95% CI)^c^	35.2 (22.5 to 47.8)	27.8 (13.1 to 42.4)	67.8 (58.8 to 76.8)	4.7 (1.7 to 7.7)	33.9 (17.7 to 50.0)	−45.3 (−67.1 to −23.6)	<.001^d^

^a^ Excludes cases with coingestants (ie, only includes cannabis exposures alone).

^b^ States with legal adult use in 2019 included: Alaska, California, Colorado, Illinois, Massachusetts, Maine, Michigan, Nevada, Oregon, Vermont, Washington State, and Washington, DC.^6^

^c^ Quarterly slope coefficients are from a linear regression of counts per quarter.

^d^ *P* value is for an interaction term between time and manufactured products group in a difference-in-difference model.

Manufactured product exposure cases more often involved children: 2505 cases (27.0%) involved patients under 10 years old, compared with 1490 plant-based exposures (8.4%). Exposures to edibles had the greatest proportion of children (1905 exposures [36.6%]). More than half of all calls were made from a health care facility; manufactured product calls came from a residence nearly twice as often as plant-based exposure calls (3927 [39.8%] vs 3817 [20.3%]; *P* < .001).

Most manufactured cannabis product exposures were for those products alone (8040 exposures [81.5%]). In contrast, most plant exposures (11 556 [61.6%]) also involved other agents (eg, alcohol, other drugs). Among exposures where only cannabis was involved, a slightly smaller percentage of manufactured product exposures overall (2918 [36.3%]) were associated with serious medical outcomes compared with plant-based exposures (2803 [38.9%]). Vaporized liquid exposures were most likely to have serious medical outcomes (268 [42.3%]).

During 2019, population-based rates for manufactured cannabis product exposures overall and for most specific products were greater where adult cannabis use was legal. One exception was vaping: exposure calls per 100 000 population were 0.29 in nonlegal states (692 total exposures) and 0.21 in legal states (195 total exposures).

Total cannabis exposures increased between 2017 and 2019. However, quarterly plant-related exposure reports declined over time, while manufactured product exposure reports increased overall and for each specific product (Figure).

**Figure.**
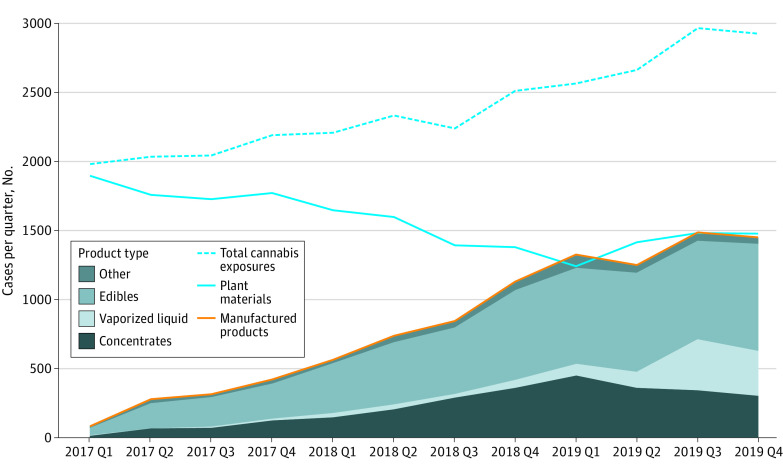
Quarterly Number of Cannabis-Related Poison Center Calls by Product Type, January 2017 to December 2019

## Discussion

Our findings document that US poison centers are increasingly receiving calls about adverse events associated with exposures to manufactured cannabis products. Higher rates in legal states suggest that continued increases may be expected with adult cannabis use legalization in more states.

Children may be at particular risk for exposure to edible products, such as cookies or candy. Although we did not see more serious health outcomes for manufactured product exposures compared with plant products overall, most cannabis plant exposures involved polysubstance use, whereas most cases for manufactured products were for those products alone, suggesting that exposure to manufactured products alone may be relatively more likely to generate adverse events. This is consistent with studies of acute health effects.^3^

Manufactured products may present risks both because of THC levels and other processing ingredients. For example, vaporized liquid additive ingredients were implicated in a 2019 national lung injury (e-cigarette or vaping use–associated lung injury [EVALI]) outbreak.^5^

 Market factors may drive the industry to continue developing novel products, which could present additional health risks. Applying regulatory controls to market-driven innovations in potency and additives is key. Novice cannabis users are often advised to “start low, go slow”; this guidance may be equally applicable to regulating new retail cannabis markets and products.

This study was limited by its data source. Poison centers provide useful information about specific product exposures and medical outcomes; however, data are self-reported and may underestimate the burden of cases. Ongoing monitoring of manufactured product–specific adverse events is recommended to understand public health concerns and effectiveness of regulations or harm reduction messaging.

## Notes

Supplement. eMethods.

## References

[1] Firth CL, Davenport S, Smart R, Dilley JA. How high: differences in the developments of cannabis markets in two legalized states. Int J Drug Policy. 2020;75:102611. doi:10.1016/j.drugpo.2019.102611 31786435 PMC7041961

[2] Smart R, Caulkins JP, Kilmer B, Davenport S, Midgette G. Variation in cannabis potency and prices in a newly legal market: evidence from 30 million cannabis sales in Washington state. Addiction. 2017;112(12):2167-2177. doi:10.1111/add.13886 28556310 PMC5673542

[3] Spindle TR, Cone EJ, Schlienz NJ, . Acute effects of smoked and vaporized cannabis in healthy adults who infrequently use cannabis: a crossover trial. JAMA Netw Open. 2018;1(7):e184841. doi:10.1001/jamanetworkopen.2018.4841 30646391 PMC6324384

[4] Matheson, J, Le Foll B. Cannabis legalization and acute harm from high potency cannabis products: a narrative review and recommendations for public health. Front Psychiatry. 2020;11:591979. doi:10.3389/fpsyt.2020.591979 PMC7538627 33173527

[5] Krishnasamy VP, Hallowell BD, Ko JY, ; Lung Injury Response Epidemiology/Surveillance Task Force. Update: characteristics of a nationwide outbreak of e-cigarette, or vaping, product use-associated lung injury—United States, August 2019-January 2020. MMWR Morb Mortal Wkly Rep. 2020;69(3):90-94. doi:10.15585/mmwr.mm6903e2 31971931 PMC7367698

[6] US Census. 2019 national and state population estimates—population for non-legal states. Updated December 30, 2019. Accessed April 9, 2021. https://www.census.gov/newsroom/press-kits/2019/national-state-estimates.html

