# Supplementary material for: Trends and Characteristics of Manufactured Cannabis Product and Cannabis Plant Product Exposures Reported to US Poison Control Centers, 2017-2019
Source: JAMA Netw Open. 2021 May 24;4(5):e2110925. doi: 10.1001/jamanetworkopen.2021.10925 (PMC8144922; doi:10.1001/jamanetworkopen.2021.10925)
Supplement: Supplement. — eMethods. [file jamanetwopen-e2110925-s001.pdf]

Supplemental Online Content

Dilley JA, Graves JM, Brooks-Russell A, Whitehill JM, Liebelt EL. Trends and characteristics of manufactured cannabis product and cannabis plant product exposures reported to US poison control centers, 2017-2019. *JAMA Netw Open.* 2021;4(5):e2110925. doi:10.1001/jamanetworkopen.2021.10925

eMethods.

This supplemental material has been provided by the authors to give readers additional information about their work.

© 2021 Dilley JA et al. *JAMA Network Open.*

eMethods.

Case definitions and exclusions.

Cannabis-related exposures were identified from the National Poison Data System (NPDS) using the following generic codes (i.e., codes with no reported manufacturer): “plant materials” include dried plant 0083000 (n=18,437) and undried plant (0310123, n=326); “manufactured products” include concentrates (0310124, n=2,734), vape liquids (vaporizer liquid/unknown if flavored 0310096 [n=779], vaporizer liquid with flavoring 0310034 [n=42], vaporizer liquor without flavoring 0310033 [n=38], marijuana liquid flavor unknown 0310097 [n=160], marijuana liquid with flavor 0310036 [n=32], marijuana liquid without flavor 0310035 [n=24]), edibles (0310121, n=5,537), and other cannabis products (capsule/pill 0310122 [n=139], pharmaceutical 0200618 [n=321], topical 0310125 [n=61]).

We excluded codes for cannabidiol (CBD, 0310146, n=1,139), unknown preparation (0310126, n=2,421), and synthetic cannabinoids (0200617, n=5,117). We also excluded duplicated exposures (i.e., if more than one cannabis type involved the second product exposure was dropped, n=221), and exposures with final Medical Outcomes of “Confirmed nonexposure” (n=64) or "Unrelated effect, the exposure was probably not responsible for the effect(s)" (n=839).

Analyses.

We examined patterns of cannabis-related exposures for specific types of manufactured cannabis products, for all manufactured cannabis products combined, and for plant-based cannabis products. We used descriptive statistics to assess patterns by year, age of person exposed, location that the exposure was reported from, and whether other substances than cannabis were involved in the exposure.

© 2021 Dilley JA et al. *JAMA Network Open.*

We characterized the final Medical Outcome for cases with no coingestants (i.e., cannabis exposures alone) in order to isolate effects of cannabis products. We characterized the following NPDS-assigned outcomes as “Moderate or greater”: *Moderate; Major; Death;* and *Unable to follow, judged as a potentially toxic exposure*. Effects of this level would usually require some form of medical treatment. “Minor or less” outcomes included: *minor effect; no effect; not followed, judged as nontoxic exposure (clinical effects not expected);* and *not followed, minimal clinical effects possible (no more than minor effect possible).*

For 2019, we compared the combined number of cases in states with legal adult cannabis use to cases in states that had not legalized adult use (this included states with legal medical cannabis). One of the states characterized as legal (Illinois) passed legalization mid-year by legislation, but no other state made substantive changes to their cannabis legalization status during this year. We excluded 126 exposures from the state analysis because state was unknown (n=34) or refused to give (n=35), or exposure occurred outside of states and D.C. For descriptive purposes, cases were also reported as rates per 100,000 population, using 2019 U.S. state population estimates.[i]

We used chi-squared tests to assess associations between manufactured cannabis products combined or plant-based cannabis exposures and exposure characteristics. We used linear regression to evaluate trends in exposure counts per quarter, and a difference-in-differences model approach to formally test for differences between slopes for manufactured and plant-based cannabis products.

All statistical analyses were conducted with Stata version 14.1 (StataCorp LP, College Station, TX) using the .05 level of significance.

© 2021 Dilley JA et al. *JAMA Network Open.*

[i] U.S. Census. Accessed at https://www.census.gov/newsroom/press-kits/2019/national-state-estimates.html NST-EST2019-01, Table 1: population for non-legal states =235,099,102; population for legal states and Washington DC =93,140,421.

© 2021 Dilley JA et al. *JAMA Network Open.*
